# Compatibility and Conjugacy on Partial Arrays

**DOI:** 10.1155/2016/5010316

**Published:** 2016-09-28

**Authors:** S. Vijayachitra, K. Sasikala

**Affiliations:** ^1^Department of Science and Humanity, Sathyabama University, Chennai, India; ^2^Department of Mathematics, St. Joseph's College of Engineering, Chennai, India

## Abstract

Research in combinatorics on words goes back a century. Berstel and Boasson introduced the partial words in the context of gene comparison. Alignment of two genes can be viewed as a construction of two partial words that are said to be compatible. In this paper, we examine to which extent the fundamental properties of partial words such as compatbility and conjugacy remain true for partial arrays. This paper studies a relaxation of the compatibility relation called *k*-compability. It also studies *k*-conjugacy of partial arrays.

## 1. Introduction

The genetic information in almost all organisms is carried by molecules of DNA. A DNA molecule is a quite long but finite string of nucleotides of 4 possible types: *a* (for adenine), *c* (for cytosine), *g* (for guanine), and *t* (for thymine). The stimulus for recent works on combinatorics is the study of biological sequences such as DNA and protein that play an important role in molecular biology [1–3]. Sequence comparison is one of the primitive operations in molecular biology. Alignment of two sequences is to place one sequence above the other [2, 4] in order to make clear correspondence between similar letters or substrings of the sequences. Partial words appear in comparing genes. Indeed, alignment of two strings can be viewed as a construction of two partial words that are compatible. The compatibility relation [5] considers two arrays with only few isolated insertions (or deletions). In some cases, it allows insertion of letters which relate to errors or mismatches. A problem appears when the same gene is sequenced by two different labs that want to differentiate the gene expression. Also, when the same long sequence is typed twice into the computer, errors appear in typing.

Partial array *A* of size (*m*, *n*) over Σ, a finite alphabet, is partial function *A* : *Z*_+_^2^ → Σ, where *Z*_+_ is the set of all positive integers. In this paper, we extend the combinatorial properties of partial words to partial arrays. Also, this paper studies a relation called *k*-compatibility where a number of insertions and deletions are allowed as well as *k*-mismatches. The conjugacy result [6] which was proved for partial words is extended to partial arrays. *k*-Conjugacy of partial arrays is discussed.

## 2. Preliminaries on Partial Words

In this section, we give a brief overview of partial words [7].

**Definition 1 .**

Partial word *u* of length *n* over *A*, a nonempty finite alphabet, is partial map *u* : {1,2,…, *n*} → *A*. If 1 ≤ *i* ≤ *n*, then *i* belongs to the domain of *u* (denoted by Domain(*u*)) in the case where *u*(*i*) is defined, and *i* belongs to the set of holes of *u* (denoted by Hole(*u*)), otherwise.

A word [8–10] is a partial word over *A* with an empty set of holes.

**Definition 2 .**

Let *u* be a partial word of length *n* over *A*. The companion of *u* (denoted by *u*_◊_) is map *u*_◊_ : {1,2,…, *n*} → *A* ∪ {◊} defined by

(1) $$\begin{array}{c} u_{◊}\left(\;i\;\right)=\left\{\begin{array}{cc} u\left(i\right) & if\;\;i\in Domain\left(u\right)\\ ◊ & otherwise. \end{array}\right. \end{array}$$

The symbol ◊ is viewed as a “do not know” symbol. Word *u*_◊_ = *ba*◊*ab*◊ is the companion of the partial word. The length of the partial word is 6. *D*(*u*) = {1,2, 4,5}. *H*(*u*) = {3,6}.

Let *u* and *v* be two partial words of length *n*. Partial word *u* is said to be contained in partial word *v* (denoted by *u* ⊂ *v*), if Domain(*u*) ⊂ Domain(*v*) and *u*(*i*) = *v*(*i*) for all *i* ∈ Domain(*u*). Partial words *u* and *v* are called compatible (denoted by *u*↑*v*), if there exists partial word *w* such that *u* ⊂ *w* and *v* ⊂ *w* (in which case we define *u*∨*v* by *u* ⊂ *u*∨*v* and *v* ⊂ *u*∨*v* and Domain(*u*∨*v*) = Domain(*u*) ∪ Domain(*v*)). As an example, *u*_◊_ = *aba*◊◊*a* and *v*_◊_ = *abab*◊*a*.

The following rules are useful for computing with partial words:

(i) *Multiplication*: If *u*↑*v* and *x*↑*y*, then *ux*↑*vy*.
(ii) *Simplification*: If *ux*↑*vy* and |*u*| = |*v*|, then *u*↑*v* and *x*↑*y*.
(iii) *Weakening*: If *u*↑*v* and *w* ⊂ *u*, then *w*↑*v*.

**Lemma 3 .**

Let *u*, *v*, *x*, *y* be partial words such that *ux*↑*vy*.

(i) If |*u* | ≥|*v*|, then there exist partial words *w*, *z* such that *u* = *wz*, *v*↑*w*, and *y*↑*zx*.
(ii) If |*u* | ≤|*v*|, then there exist partial words *w*, *z* such that *b* = *wz*, *v*↑*w*, and *x*↑*zy*.

**Definition 4 .**

Two partial words *u* and *v* are called conjugate, if there exist partial words *x* and *y* such that *u* ⊂ *xy* and *v* ⊂ *yx*.

**Definition 5 .**

Two partial words *u* and *v* are called *k*-conjugate, if there exist nonnegative integers *k*_1_, *k*_2_ whose sum is *k* and partial words *x* and *y* such that *u*⊂_*k*_1__ *xy* and *v*⊂_*k*_2__ *yx*.

## 3. Preliminaries on Partial Arrays

This section is devoted to review the basic concepts on partial arrays [11].

**Definition 6 .**

Partial array *A* of size (*m*, *n*) over Σ, a nonempty set or an alphabet, is partial function *A* : *Z*_+_^2^ → Σ, where *Z*_+_ is the set of all positive integers. For 1 ≤ *i* ≤ *m*, 1 ≤ *j* ≤ *n*, and if *A*(*i*, *j*) is defined, then we say that (*i*, *j*) belongs to the domain of *A* (denoted by (*i*, *j*) ∈ *D*(*A*)). Otherwise, we say that (*i*, *j*) belongs to the set of holes of *A* (denoted by (*i*, *j*) ∈ *H*(*A*)).

An array [5] over Σ is a partial array over Σ with an empty set of holes.

**Definition 7 .**

If *A* is a partial array of size (*m*, *n*) over Σ, then the companion of *A* (denoted by *A*_◊_) is total function *A*_◊_ : *Z*_+_^2^ → Σ ∪ {◊} defined by

(2) $$\begin{array}{c} A_{◊}\left(\;i,j\;\right)=\left\{\begin{array}{cc} A\left(i,j\right) & if\;\;\left(i,j\right)\in D\left(A\right)\\ ◊ & otherwise, \end{array}\right. \end{array}$$

where ◊∉Σ.

The bijectivity of map *A* → *A*_◊_ allows defining the catenation of two partial arrays in a trivial way.

**Example 8 .**

Partial array $A=\left(\begin{array}{ccc} b & a & b\\ ◊ & a & b\\ b & ◊ & b \end{array}\right)$ is the companion of partial array *A* of size (3, 3), where *D*(*A*) = {(1,1), (1,2), (1,3), (2,2), (2,3), (3,1), (3,3)}, *H*(*A*) = {(2,1), (3,2)}.

Let

(3) X=a11⋯a1n⋮⋮am1⋯amn,Y=b11⋯b1n′⋮⋮bm′1⋯bm′n′.

By column catenation, we mean

(4) $$\begin{array}{c} X\;⦶\;Y=\left(\begin{array}{cccccc} a_{11} & \cdots & a_{1n} & b_{11} & \cdots & b_{1n^{\prime }}\\ \vdots & & \vdots & \vdots & & \vdots \\ a_{m1} & \cdots & a_{mn} & b_{m^{\prime }1} & \cdots & b_{m^{\prime }n^{\prime }} \end{array}\right). \end{array}$$

By row catenation, we mean

(5) $$\begin{array}{c} X⊖Y=\left(\begin{array}{ccc} a_{11} & \cdots & a_{1n}\\ \vdots & & \vdots \\ a_{m1} & \cdots & a_{mn}\\ b_{11} & \cdots & b_{1n^{\prime }}\\ \vdots & & \vdots \\ b_{m^{\prime }1} & \cdots & b_{m^{\prime }n^{\prime }} \end{array}\right). \end{array}$$

If *A* and *B* are two partial arrays of equal size, then *A* is contained in *B* denoted by *A* ⊂ *B* if *D*(*A*)⊆*D*(*B*) and

(6) $$\begin{array}{c} A\left(\;i,j\;\right)=B\left(\;i,j\;\right)\;\forall \left(i,j\right)\in D\left(A\right). \end{array}$$

**Definition 9 .**

Partial arrays *A* and *B* are said to be compatible denoted by *A*↑*B*, if there exists partial array *C* such that *A* ⊂ *C* and *B* ⊂ *C*.

## 4. Compatibiltiy and *k*-Compatability of Partial Arrays

### 4.1. Compatibility

The rules mentioned for partial words are also true for partial arrays.

Let *A*,  *B*,  *X*,  *Y* be partial arrays.

(i) *Multiplication*: If *A*↑*B* and *X*↑*Y*, then *AX*↑*BY* either by column catenation or by row catenation.
(ii) *Simplification*: If *AX*↑*BY* either by column catenation or by row catenation with *A* and *B* being of same size, then *A*↑*B* and *X*↑*Y*.
(iii) *Weakening*: If *A*↑*B* and *C* ⊂ *A*, then *C*↑*B*.

Lemma 3's version for partial arrays can be stated as follows.

**Lemma 10 .**

Let *A*,  *B*,  *X*,  *Y* be partial arrays such that *AX*↑*BY*, either by column catenation or by row catenation.

(i) If order of *A*≥ order of *B*, then there exist partial arrays *C*, *Z* such that *A* = *CZ*, *B*↑*C*, and *Y*↑*ZX*.
(ii) If order of *A*≤ order of *B*, then there exist partial arrays *C*, *Z* such that *B* = *CZ*, *A*↑*C*, and *X*↑*ZY*.

### 4.2. *k*-Compatibility

**Definition 11 .**

If *A* and *B* are two partial arrays of same size and *k* is nonnegative integer, then *A* is said to be *k*-contained in *B* denoted by *A*⊂_*k*_ *B* if *D*(*A*) ⊂ *D*(*B*) and there exists subset *E* of *D*(*A*) of cardinality *k* called the error set such that

(7) Ai,j=Bi,j∀i,j∈DA∖E,Ai,j≠Bi,j∀i,j∈E.

**Definition 12 .**

If *A* and *B* are two partial arrays of same order and *k* is a nonnegative integer, then *A* and *B* are called *k*-compatible denoted by *A*↑_*k*_ *B* if there exist partial array *Z* and nonnegative integers *k*_1_,  *k*_2_ such that

(i) *A*⊂_*k*_1__ *Z* with error set *E*_1_;
(ii) *B*⊂_*k*_2__ *Z* with error set *E*_2_;
(iii) *E*_1_∩*E*_2_ = *ϕ*;
(iv) *k*_1_ + *k*_2_ = *k*.

**Example 13 .**

$A=\left(\begin{array}{ccc} b & a & b\\ a & ◊ & c \end{array}\right)$, $B=\left(\begin{array}{ccc} a & b & a\\ a & ◊ & c \end{array}\right)$, then there exists partial array $Z=\left(\begin{array}{ccc} a & b & b\\ a & ◊ & c \end{array}\right)$ with *E*_1_ = {(1,1), (1,2)}, *E*_2_ = {(1,3)} and *k*_1_ = 2, *k*_2_ = 1⇒*k* = 3; that is, *A*↑_3_ *B*.

Equivalently, *A* and *B* are *k*-compatible, if there exists subset *E* of *D*(*A*)∩*D*(*B*) of cardinality *k* called the error set such that

(i) *A*(*i*, *j*) = *B*(*i*, *j*)∀(*i*, *j*) ∈ *D*(*A*)∩*D*(*B*)∖*E*;
(ii) *A*(*i*, *j*) ≠ *B*(*i*, *j*)∀(*i*, *j*) ∈ *E*.

If *A* and *B* are arrays, then *A*↑_∘_ *B* means *A* = *B*. We sometimes use notation *A*↑_≤*k*_ *B*, if set *E* has cardinality ≤*k*.

*Multiplication*. If *A*↑_*k*_1__ *B* and *X*↑_*k*_2__ *Y*, then *AX*↑_*k*_1_+*k*_2__ *BY* where *A*,  *B*,  *X*, and *Y* are partial arrays and *k*_1_,  *k*_2_ are nonnegative integers, using column catenation.

**Example 14 .**

$A=\left(\begin{array}{ccc} ◊ & a & a\\ b & b & ◊\\ a & b & a \end{array}\right)$, $B=\left(\begin{array}{ccc} b & b & ◊\\ a & a & ◊\\ a & a & b \end{array}\right)$, $X=\left(\begin{array}{ccc} b & ◊ & a\\ a & b & a\\ a & ◊ & b \end{array}\right)$, $Y=\left(\begin{array}{ccc} a & b & b\\ b & a & b\\ ◊ & ◊ & a \end{array}\right)$.

*AX*↑_6+7_ *BY*.

*Simplification.* If *AX*↑_*k*_ *BY* and order of *A* is equal to order of *B*, then *A*↑_*k*_1__ *B* and *X*↑_*k*_2__ *Y* for some *k*_1_,  *k*_2_, satisfying *k*_1_ + *k*_2_ = *k*.

**Example 15 .**

$A=\left(\begin{array}{ccc} ◊ & a & a\\ b & b & ◊\\ a & b & a \end{array}\right)$, $B=\left(\begin{array}{ccc} b & b & ◊\\ a & ◊ & a\\ b & a & b \end{array}\right)$, $X=\left(\begin{array}{cc} b & ◊\\ a & b\\ a & ◊ \end{array}\right)$, $Y=\left(\begin{array}{c} a\\ b\\ b \end{array}\right)$.

*AX*↑_8_ *BY*⇒*A*↑_5_ *B* and *X*↑_3_ *Y* with 5 + 3 = 8.

*Weakening.* If *A*↑_*k*_ *B* and *Z* ⊂ *A*, then *Z*↑_≤*k*_ *B*.

**Example 16 .**

$A=\left(\begin{array}{ccc} ◊ & a & a\\ b & b & ◊\\ a & b & a \end{array}\right)$, $B=\left(\begin{array}{ccc} b & b & ◊\\ ◊ & a & ◊\\ b & a & b \end{array}\right)$, $Z=\left(\begin{array}{cc} a & a\\ b & ◊\\ b & a \end{array}\right)$.

*Z*↑_≤7_ *B* with *k* = 7.

**Theorem 17 .**

Let *A* and *B* be partial arrays of orders *a* × *b* and *a* × *c*, respectively. If there exist array *Z* of order *a* × *d* and integers *k*_1_,  *k*_2_,  *m*, and *n* such that *A*⊂_*k*_1__ *Z* ^*m*^ with error set *E*_1_ and *B*⊂_*k*_2__ *Z* ^*n*^ with error set *E*_2_, then there exist integers *p* and *q* such that *A* ^*p*^↑_≤*k*_ *B* ^*q*^ with

(8) k=DAa,b,p∩E2a,c,q∪DBa,c,q∩E1a,b,p.

Moreover, if *E*_1_(*a*, |*b*|, *n*)∩*E*_2_(*a*, |*c*|, *m*) = *ϕ*, then *A* ^*p*^↑_*k*_ *B* ^*q*^.

**Proof**

Let *A* and *B* be partial arrays of *a* × *b* and *a* × *c*, respectively. Let array *z* of order *a* × *d* exist such that, by using column catenation, *A*⊂_*k*_1__ *Z* ^*m*^ and *B*⊂_*k*_2__ *Z* ^*n*^ for some integers *k*_1_,  *k*_2_,  *m*, and *n*. Let *E*_1_ be the error set of cardinality *k*_1_ such that *A*(*i*, *j*) = *Z* ^*m*^(*i*, *j*) for all (*i*, *j*) ∈ *D*(*A*)∖*E*_1_ and *A*(*i*, *j*) ≠ *Z* ^*m*^(*i*, *j*) for all (*i*, *j*) ∈ *E*_1_ and *E*_2_ be the error set of cardinality *k*_2_ such that *B*(*i*, *j*) = *Z* ^*n*^(*i*, *j*) for all (*i*, *j*) ∈ *D*(*B*)∖*E*_2_ and *B*(*i*, *j*) ≠ *Z* ^*n*^(*i*, *j*) for all (*i*, *j*) ∈ *E*_2_. We have *A*_*n*_⊂_*nk*_1__ *Z* ^*mn*^ with error set *E*_1_(*a*, |*b* | , *n*) of cardinality *nk*_1_ and *B* ^*m*^⊂_*mk*_2__ *Z* ^*mn*^ with error set *E*_2_(*a*, |*c* | , *m*) of cardinality *mk*_2_.

Let (1,1)≤(*i*, *j*)≤(*a*, *d* ^*mn*^) and *Z* ^*mn*^(*i*, *j*) = *a* for some letter *a*. There are 4 possibilities.

*Case 1*. If (*i*, *j*) ∉ *E*_1_(*a*, |*b* | , *n*) and (*i*, *j*) ∉ *E*_2_(*a*, |*c* | , *m*), then *A* ^*n*^(*i*, *j*)∈{◊, *a*} and *B* ^*m*^(*i*, *j*)∈{◊, *a*}. It does not give any error, when we align *A* ^*n*^ with *B* ^*m*^.

*Case 2*. If (*i*, *j*) ∉ *E*_1_(*a*, |*b* | , *n*) and (*i*, *j*) ∈ *E*_2_(*a*, |*c* | , *m*), then *A* ^*n*^(*i*, *j*)∈{◊, *a*} and *B* ^*m*^(*i*, *j*) = *b* for some *b* ≠ *a*. It gives an error in the alignment of *A* ^*n*^ with *B* ^*m*^ only when *A* ^*n*^(*i*, *j*) = *a* or when (*i*, *j*) ∈ *D*(*A*)(*a*, |*b* | , *n*).

*Case 3*. If (*i*, *j*) ∈ *E*_1_(*a*, |*b* | , *n*) and (*i*, *j*) ∈ *E*_2_(*a*, |*c* | , *m*), then *B* ^*m*^(*i*, *j*)∈{◊, *a*} and *A* ^*n*^(*i*, *j*) = *b* for some *b* ≠ *a*. It gives an error in the alignment of *A* ^*n*^ with *B* ^*m*^ only when *B* ^*m*^(*i*, *j*) = *a* or when (*i*, *j*) ∈ *D*(*B*)(*a*, |*c* | , *m*).

*Case 4*. If (*i*, *j*) ∈ *E*_1_(*a*, |*b* | , *n*) and (*i*, *j*) ∈ *E*_2_(*a*, |*c* | , *m*), then *A* ^*n*^(*i*, *j*) = *b* for some *b* ≠ *a* and *B* ^*m*^(*i*, *j*) = *c* for some *c* ≠ *a*. It gives an error in the alignment of *A* ^*n*^ with *B* ^*m*^ only when *b* ≠ *c*.

Therefore, if *E*_1_(*a*, |*b* | , *n*)∩*E*_2_(*a*, |*c* | , *m*) = *ϕ* then *A* ^*n*^↑_*k*_ *B* ^*m*^ with *k* = ‖(*D*(*a*)(*a*, |*b* | , *n*)∩*E*_2_(*a*, |*c* | , *m*))∪(*D*(*B*)(*a*, |*c* | , *m*)∩*E*_1_(*a*, |*b* | , *n*)‖ and *E*_1_(*a*, |*b* | , *n*)∩*E*_2_(*a*, |*c* | , *m*) ≠ *ϕ* then *A* ^*n*^↑_≤*k*_ *B* ^*m*^.

**Example 18 .**

$A=\left(\begin{array}{ccc} a & b & ◊\\ c & a & b\\ ◊ & b & a \end{array}\right)$, $B=\left(\begin{array}{cc} a & c\\ c & b\\ ◊ & b \end{array}\right)$, $Z=\left(\begin{array}{c} a\\ c\\ b \end{array}\right).$

We have *A*⊂_4_ *Z* ^3^ with error set *E*_1_ = {(1,2), (2,2), (2,3), (3,3)}, and  *B*⊂_2_ *Z* ^2^ with error set *E*_2_ = {(1,2), (2,2)}.

 
*k* = 6:
  (i) *D*(*A*) = {(1,1), (1,2), (2,1), (2,2), (2,3), (3,2),(3,3)}.
   
*D*(*B*) = {(1,1), (1,2), (2,1), (2,2), (3,2)}
  (ii) *D*(*A*)(*a*, |*b*|, 2) = {(1,1), (1,2), (2,1), (2,2), (2,3), (3,2), (3,3), (1,4), (1,5), (2,4), (2,5), (2,6), (3,5), (3,6)}.
   
*D*(*B*)(*a*, |*c*|, 3) = {(1,1), (1,2), (2,1), (2,2), (3,2), (1,3), (1,4), (2,3), (2,4), (3,4), (1,5), (1,6), (2,5), (2,6), (3,6)}
  (iii) *E*_1_(*a*, |*b*|, 2) = {(1,2), (2,2), (2,3), (3,3), (1,5), (2,5), (2,6), (3,6)}.
   
*E*_2_(*a*, |*c*|, 3) = {(1,2), (2,2), (1,4), (2,4), (1,6), (2,6)}.
   
*E*_1_(*a*, |*b*|, 2)∩*E*_2_(*a*, |*c*|, 3) ≠ *ϕ*.
 
*k* = ‖(*D*(*A*)(*a*, |*b*|, 2)∩*E*_2_(*a*, |*c*|, 3)) ∪ (*D*(*B*)(*a*, |*c*|, 3)∩*E*_1_(*a*, |*b*|, 2))‖ = ‖(((1,1), (1,2), (2,1), (2,2), (2,3), (3,2), (3,3), (1,4), (1,5), (2,4), (2,5), (2,6), (3,5), (3,6))∩((1,2), (2,2), (1,4), (2,4), (1,6), (2,6)))∪(((1,1), (1,2), (2,1), (2,2), (3,2), (1,3), (1,4), (2,3), (2,4), (3,4), (1,5), (1,6), (2,5), (2,6), (3,6))∩((1,2), (2,2), (2,3), (3,3), (1,5), (2,5), (2,6), (3,6)))‖ = ‖(1,2), (1,4), (1,5), (2,2), (2,3), (2,4), (2,5), (2,6), (3,6)‖.
 
*k* = 9:

*A* ^2^↑_≤9_ *B* ^3^(*A* ^2^↑_6_ *B* ^3^).

## 5. Conjugacy and *k*-Conjugacy of Partial Arrays

### 5.1. Conjugacy

**Definition 19 .**

Two partial arrays *A* and *B* of same order are called conjugate if there exist partial arrays *X* and *Y* such that *A* ⊂ *XY* and *B* ⊂ *YX* using row catenation or column catenation.

0-conjugacy on partial arrays with same order is trivially reflexive and symmetric but not transitive.

**Example 20 .**

$A=\left(\begin{array}{ccc} a & ◊ & b\\ b & c & a\\ a & a & ◊ \end{array}\right)$, $B=\left(\begin{array}{ccc} b & c & ◊\\ a & a & ◊\\ a & c & b \end{array}\right)$, $C=\left(\begin{array}{ccc} b & a & ◊\\ b & a & ◊\\ a & b & c \end{array}\right)$.

By taking $X=\left(\begin{array}{ccc} a & c & b \end{array}\right)$ and $Y=\left(\begin{array}{ccc} b & c & a\\ a & a & ◊ \end{array}\right)$, we get *A* ⊂ *XY* and *B* ⊂ *YX* showing that *A* and *B* are conjugate similarly and, by taking $X^{\prime }=\left(\begin{array}{ccc} b & c & ◊ \end{array}\right)$ and $Y^{\prime }=\left(\begin{array}{ccc} a & c & b \end{array}\right)$, we get *B* ⊂ *X*′*Y*′ and *C* ⊂ *Y*′*X*′ showing that *B* and *C* are conjugate. But *A* and *C* are not conjugate.

That is, conjugate relation is not transitive.

**Proposition 21 .**

Let *A* and *B* be nonempty partial arrays of same size. If *A* and *B* are conjugate, then there exists partial array *C* such that *AC*↑*CB*, either by column catenation or by row catenation.

**Proof**

Let *A* and *B* be nonempty partial arrays of same order. Suppose *A* and *B* are conjugate and let *X*,  *Y* be partial arrays such that *A* ⊂ *XY* and *B* ⊂ *YX* either by column catenation or by row catenation; then *AX* ⊂ *XYX* and *XB* ⊂ *XYX*. So, for *C* = *X*, we have *AC*↑*CB*.

### 5.2. *k*-Conjugacy

**Definition 22 .**

Two partial arrays *A* and *B* of same order are *k*-conjugate, if there exist nonnegative integers *k*_1_ *k*_2_ whose sum is *k* and partial arrays *X* and *Y* such that *A*⊂_*k*_1__ *XY* and *B*⊂_*k*_2__ *YX* with row or column catenation.

**Theorem 23 .**

Let *A* and *B* be nonempty partial arrays of same order. If *A* and *B* are *k*-conjugate, then there exists partial array *Z* such that *AZ*↑_≤*k*_ *ZB*.

**Proof**

Let *A*,  *B* be two partial arrays of same order. Supposing that *A* and *B* are *k*-conjugate, then, by definition, there exist nonnegative integers *k*_1_, *k*_2_ whose sum is *k* and partial arrays *X* and *Y* such that *A*⊂_*k*_1__ *XY* with error set *E*_1_ and *B*⊂_*k*_2__ *YX* with error set *E*_2_ using row catenation or column catenation accordingly.

Then, *AX*⊂_*k*_1__ *XYX* with error set *E*_1_ and *XB*⊂_*k*_2__ *XYX* with error set *E*_2_′ = {(*i* + number  of  rows  of  *X*, *j*)/(*i*, *j*) ∈ *E*_2_} or *E*_2_′ = {(*i*, *j* + number  of  columns  of  *X*)/(*i*, *j*) ∈ *E*_2_} according to row or column catenation and so, for *Z* = *X*, we have *AZ*↑_≤*k*_ *ZB*.

**Example 24 .**

Given $A=\left(\begin{array}{ccc} a & ◊ & b\\ b & c & a\\ a & a & ◊ \end{array}\right)$, $B=\left(\begin{array}{ccc} b & c & b\\ a & a & ◊\\ a & ◊ & b \end{array}\right)$.

There exist $X=\left(\begin{array}{ccc} a & ◊ & b \end{array}\right)$ and $Y=\left(\begin{array}{ccc} c & a & b\\ a & a & ◊ \end{array}\right)$ with *A*⊂_3_ *XY* and *B*⊂_2_ *YX*, *k* = *k*_1_ + *k*_2_ = 5.

There exist $Z=\left(\begin{array}{ccc} a & ◊ & b \end{array}\right)$ such that *AZ*↑_≤5_ *ZB*.

## 6. Conclusion

Motivated by compatibility and conjugacy properties of partial words, we define the conjugacy of partial array and derive the compatibility properties of partial arrays. By giving relaxation to the compatibility relation, we discuss *k*-compatibility and *k*-conjugacy of partial arrays. We prove that, given partial arrays *A*,  *B* and integers *p*,  *q* satisfying |*A*|^*p*^ = |*B*|^*q*^, we find *k* such that *A* ^*p*^↑_*k*_ *B* ^*q*^. Also, there exists partial array *Z* such that *AZ*↑_≤*k*_ *ZB*.

## References

[1] Arquès D. G., Michel Ch. J. (1995). A possible code in the genetic code. *STACS 95*.

[2] Blanchet-Sadri F., Hegstrom R. A. (2002). Partial words and a theorem of Fine and Wilf revisited. *Theoretical Computer Science*.

[3] Colosimo A., de Luca A. (2000). Special factors in biological strings. *Journal of Theoretical Biology*.

[4] Blanchet-Sadri F., Dakota Blair D., Lewis R. V. (2009). Equations on partial words. *RAIRO—Theoretical Informatics and Applications*.

[5] Sweety F., Thomas D. G., Kalyani T., Bebis G., Boyle R., Parvin B. (2008). Collage of hexagonal arrays. *Advances in Visual Computing*.

[6] Blanchet-Sadri F., Luhmann D. K. (2002). Conjugacy on partial words. *Theoretical Computer Science*.

[7] Berstel J., Boasson L. (1999). Partial words and a theorem of Fine and Wilf. *Theoretical Computer Science*.

[8] de Luca A. (1999). On the combinatorics of finite words. *Theoretical Computer Science*.

[9] Choffrut C., Karthumaki J., Rozenberg G., Salomaa A. (1999). Combinatorics of words. *Handbook of Formal Languages*.

[10] Lothaire M. (1997). *Combinatorics on Words*.

[11] Sweety F., Thomas D. G., Dare V. R. (2006). Subarray complexity of partial arrays. *Intelligent Optimization Modeling*.

